# Effect of preoperative oral antibiotics in combination with mechanical bowel preparation on inflammatory response and short‐term outcomes following left‐sided colonic and rectal resections

**DOI:** 10.1002/bjs5.50224

**Published:** 2019-10-16

**Authors:** A. M. Golder, C. W. Steele, D. Conn, G. J. MacKay, D. C. McMillan, P. G. Horgan, C. S. Roxburgh, S. T. McSorley

**Affiliations:** ^1^ Academic Unit of Surgery, Glasgow Royal Infirmary Glasgow UK

## Abstract

**Background:**

Preoperative oral antibiotics in addition to intravenous antibiotics and mechanical bowel preparation (MBP) may influence the gut microbiome and reduce both the postoperative systemic inflammatory response to surgery and postoperative infective complications following colorectal resection. This propensity score‐matched study compared outcomes of patients undergoing left‐sided colonic or rectal resection with or without a combination of oral antibiotics and MBP.

**Methods:**

The addition of oral antibiotics and MBP to prophylactic intravenous antibiotics in left‐sided colonic and rectal resections was introduced in 2015–2016 at a single institution. Propensity score matching was undertaken to compare the effects of oral antibiotics plus MBP *versus* neither oral antibiotics nor MBP on the postoperative systemic inflammatory response and short‐term outcomes in patients undergoing left‐sided colonic or rectal resection between 2013 and 2018.

**Results:**

Of 396 patients who had propensity score matching for host, anaesthetic and operative factors, 204 matched patients were identified. The addition of oral antibiotics and MBP was associated with a significantly reduced postoperative inflammatory response (reduced postoperative Glasgow Prognostic Score) on day 3 (odds ratio (OR) 0·66, 95 per cent c.i. 0·44 to 0·99; *P* = 0·013) and day 4 (OR 0·46, 0·30 to 0·71; *P* = 0·001). Significantly reduced overall complications (OR 0·31, 0·17 to 0·56; *P* < 0·001), infective complications (OR 0·41, 0·22 to 0·77; *P* = 0·011), surgical‐site infection (OR 0·37, 0·17 to 0·83; *P* = 0·024) and postoperative length of hospital stay (median 7 days *versus* 8 days in patients who had intravenous antibiotics alone; *P* = 0·050) were also observed.

**Conclusion:**

Preoperative oral antibiotics and MBP in addition to prophylactic intravenous antibiotics were associated with a reduction in the postoperative systemic inflammatory response and postoperative complications in patients undergoing resectional left‐sided colonic or rectal surgery.

## Graphical abstract

This single‐centre propensity‐matched cohort study compared postoperative outcomes in patients undergoing left‐sided colonic or rectal resection with or without preoperative oral antibiotics and mechanical bowel preparation. In 204 matched patients, the addition of oral antibiotics and mechanical bowel preparation was associated with a significant reduction in postoperative systemic inflammatory response, overall complications, infective complications, surgical‐site infection and length of hospital stay.

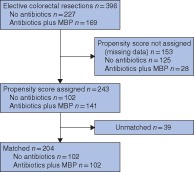

Evidence of benefit

## Abstract (es)

**Antecedentes:**

La administración preoperatoria de antibióticos por vía oral (*preoperative oral antibiotics*, OAB), además de por vía intravenosa y de la preparación mecánica del colon (*mechanical bowel preparation,* MBP) puede afectar al microbioma intestinal y disminuir tanto la respuesta postoperatoria sistémica inflamatoria a la cirugía, como las complicaciones infecciosas postoperatorias tras una resección colorrectal. Este estudio emparejado por puntaje de propensión comparó los resultados de pacientes sometidos a resección del colon izquierdo o resección rectal con y sin una combinación de OAB y MBP.

**Métodos:**

La adición de OAB y MBP a la administración profiláctica de antibióticos por vía intravenosa fue introducida en 2015‐2016 en un centro médico. Se llevó a cabo un estudio emparejado por puntaje de propensión para comparar los efectos de OAB con MBP versus la no administración de OAB ni el uso de MBP sobre la respuesta postoperatoria sistémica inflamatoria a la cirugía y los resultados a corto plazo en pacientes sometidos a resección del colon izquierdo o resección rectal desde el 2013 al 2018.

**Resultados:**

De los 396 pacientes incluidos en el emparejamiento por puntaje de propensión relativo a factores relacionados con el huésped, anestésicos y operatorios, se identificaron 204 pacientes emparejados. La adición de OAB y MBP se asoció con una disminución significativa de la respuesta inflamatoria postoperatoria (disminución postoperatoria de la puntuación pronóstica de Glasgow el día 3 (razón de oportunidades, *odds ratio*, OR 0,66, i.c. del 95% 0,44‐0,99, *P* = 0,013) y el día 4 (OR 0,46, i.c. del 95% 0,30‐0,71, *P* = 0,001). También se observaron reducciones significativas de las complicaciones globales (OR 0,31, i.c. del 95% 0,17‐0,56, *P* < 0,001), complicaciones infecciosas (OR 0,41, i.c. del 95% 0,17‐0,83, *P* = 0,011), infecciones del sitio quirúrgico (OR 0,37, i.c. del 95% 0,17‐0,83, *P* = 0,024) y duración de la estancia hospitalaria postoperatoria (mediana 8 *versus* 7 días, *P* = 0,05).

**Conclusión:**

La adición preoperatoria de OAB y MBP a la administración profiláctica de antibióticos intravenosos se han asociado con una disminución de la respuesta inflamatoria sistémica postoperatoria y de las complicaciones postoperatorias en pacientes sometidos a resección del colon izquierdo o cirugía rectal.

## Introduction

Approximately 1·8 million patients are diagnosed with colorectal cancer worldwide each year^1^. Resectional surgery remains the mainstay of treatment, albeit with significant morbidity as well as cancer‐related mortality, even after curative treatment^2^.

A previous Cochrane review^3^ reported clear evidence for antibiotic prophylaxis *versus* no antibiotic prophylaxis in the reduction of surgical‐site infection (SSI) following colorectal surgery. In that review, evidence comparing either oral or intravenous antibiotic administration was limited, although no significant difference was found. When a combination of intravenous and oral antibiotic therapy was compared with single‐route administration, a significant reduction in wound infection was reported (risk ratio 0·55, *P* < 0·001). Most studies have compared the effects of oral antibiotic administration in the context of mechanical bowel preparation (MBP). The effect of oral antibiotics in addition to intravenous antibiotics in the unprepared colon is uncertain. This is reflected in the WHO SSI prevention guidelines^4^ and a recent update to the Enhanced Recovery After Surgery (ERAS®) Society guidelines^5^, both of which support the use of intravenous antibiotic prophylaxis immediately before surgery in addition to oral antibiotics when MBP is being used, but acknowledge the need for further research into the role of oral antibiotics without MBP.

After colorectal resection there are significant relationships between increased postoperative systemic inflammatory response and more postoperative morbidity^6^, ^7^, ^8^, as well as poorer long‐term oncological outcomes^9^, ^10^, ^11^. Measures that modulate the systemic inflammatory response after surgery and reduce postoperative complications are therefore of clinical interest.

The administration of prophylactic intravenous antibiotics on induction of anaesthesia is now routine, whereas the use of preoperative oral antibiotics remains limited, as reported in a recent European survey^12^ and international audit^13^. Evidence, largely from the USA and Japan, has been summarized by several meta‐analyses^14^, ^15^, ^16^, ^17^ reporting a reduction in postoperative infective complications following the use of oral in addition to intravenous antibiotics. It has been hypothesized^18^, ^19^ that a local alteration in the gut microbiome could have a significant relationship with the host systemic inflammatory response, modifying the risk of complications.

The primary aim of this propensity‐matched study was to examine the impact of preoperative oral antibiotics in combination with MBP, given in addition to intravenous antibiotics, on postoperative infective complications in patients undergoing left‐sided colonic or rectal resection. As a secondary aim, the impact of preoperative oral antibiotics on the postoperative systemic inflammatory response was examined.

## Methods

This longitudinal observational study included patients aged at least 18 years undergoing elective left‐sided colonic or rectal resection at Glasgow Royal Infirmary between 2013 and 2018. Surgery was undertaken for either benign disease or colorectal cancer resection with curative intent. Patients with coexisting inflammatory bowel disease or metastatic disease, those undergoing multivisceral resections, and those undergoing emergency surgery were excluded. All operations were performed or supervised by a consultant colorectal surgeon.

During the time period for inclusion into this study, an ERAS pathway was in place to standardize perioperative care. This included: preoperative carbohydrate‐loading where appropriate; prophylactic intravenous antibiotics at induction of anaesthesia; venous thromboprophylaxis; early enteral nutrition; early mobilization; and avoidance of routine peritoneal or nasogastric drainage. The use of perioperative dexamethasone to reduce the risk of postoperative nausea and vomiting, and regional anaesthetic technique was at the discretion of the surgical and anaesthetic teams caring for the patient.

Preoperative oral antibiotics and MBP were introduced in 2015, but their use was not widespread until 2016. Before 2015–2016, and therefore in the control group, neither oral antibiotics nor MBP were in routine use. Instead, patients undergoing rectal surgery were given a single phosphate enema, whereas those having a left‐sided colonic resection received no bowel preparation. Patients in the treatment group were treated predominantly in 2016–2018, and received preoperative oral antibiotics in the form of neomycin (1 g) and metronidazole (400 mg) at 15·00, 16·00 and 22·00 hours on the day before surgery, and oral MBP in the form of four sachets of Klean‐Prep® (Norgine, Harefield, UK), a macrogol‐based laxative. All patients received prophylactic intravenous antibiotics on induction of anaesthesia regardless of whether they were in the control group, predominantly from 2013 to 2016, or the treatment group, predominantly from 2016 to 2018.

Patients were assessed clinically every day after surgery with blood analysis, including estimation of C‐reactive protein (CRP) on most postoperative days. Other investigations and interventions for clinical or biochemical concerns were carried out at the discretion of the team caring for the patient.

Clinicopathological data were collected in a secure, prospectively collated, electronic database in line with local NHS Greater Glasgow and Clyde policy, which detailed whether or not patients received oral antibiotics and MBP. Additional data, including laboratory results, clinical letters, inpatient records and operation notes, were obtained from online patient records.

Clinicopathological data collected included: site of surgery (left‐sided, rectal without perineal incision, rectal with perineal incision); presence of diabetes; use of perioperative dexamethasone; use of regional anaesthesia; surgical approach (laparoscopic or open); duration of surgery; need for intraoperative blood transfusion; and the preoperative inflammatory state using the modified Glasgow Prognostic Score (mGPS)^20^.

Outcomes of interest were postoperative complications, the postoperative inflammatory response, and postoperative length of hospital stay.

Complications were categorized as overall complications, infective complications and non‐infective complications. Infective complications were subcategorized as non‐SSI or SSIs, which were then further subcategorized as follows: superficial SSI (presence of pus either discharging spontaneously or requiring drainage or the use of antibiotics due to a diagnosis of cellulitis around the wound); deep SSI (intra‐abdominal pus or infection requiring either drainage or antibiotic therapy); or anastomotic leakage (diagnosed either on imaging or at laparotomy). Complications were categorized by severity using the Clavien–Dindo classification^21^.

A CRP threshold above 150 mg/l and an albumin level below 25 mg/l on day 3 and 4 was recorded. A postoperative Glasgow Prognostic Score (poGPS)^8^ was calculated for patients for whom CRP and albumin results were available. Postoperative mortality was defined as death within 30 days of surgery. Length of hospital stay was defined as the median number of days between surgery and hospital discharge.

This study was approved by NHS Greater Glasgow and Clyde Information Governance Unit and the Caldicott Guardian as part of surgical audit. Data were collected by clinical research fellows, with discussion with a senior author when appropriate. Those collecting data had access to the database and online patient records.

### Statistical analysis

In the unmatched cohort, categorical data were compared using the χ^2^ test. Data on postoperative length of stay were non‐parametric and are presented as median (range) values. Medians were compared using an independent‐samples median test. A two‐sided *P* < 0·050 was considered significant. Measurement of association between variables for both unmatched and matched data was carried out using either the φ coefficient or Cramer's V test (φ_c_). All statistical analyses were carried out using IBM SPSS® version 24 for Windows® (IBM, Armonk, New York, USA).

Patients were propensity score matched for age, sex, site of surgery, surgical approach, BMI, ASA grade, smoking status, preoperative mGPS, intraoperative blood transfusion, stoma formation, coexisting diabetes, use of regional anaesthesia, and use of preoperative dexamethasone. Each patient who received preoperative oral antibiotics was matched 1 : 1 with a patient who did not, using the closest propensity score on the logit scale (calliper less than 0·05, order of match selection randomized, without replacement). The appropriateness of propensity score matching was assessed visually by the frequency of propensity scores in each group before and after matching. Propensity scores were calculated only for patients who did not have missing data in the variables used in the propensity scoring process.

Within the propensity score‐matched data, McNemar's test was used to compare categorical data when this was in a 2 × 2 table or using a McNemar–Bowker test^22^ when the data table was more than 2 × 2. Binary logistic regression was used to calculate odds ratios (ORs) and 95 per cent c.i. for outcomes within the propensity score‐matched data set.

The treatment effect of preoperative oral antibiotics and MBP in terms of poGPS on day 3 after surgery, overall complications and overall infective complications was displayed as ORs with 95 per cent c.i. for unmatched and matched patients. In addition, propensity scores were included along with preoperative oral antibiotics and MBP as a linear co‐variable in multivariable binary logistic regression for day 3 poGPS, overall complications and infective complications.

## Results

**Figure 1:**
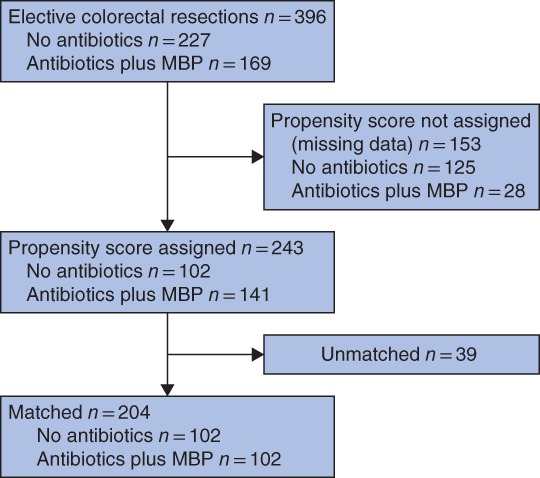
Flow diagram of antibiotic administration before and after propensity score matching MBP, mechanical bowel preparation.

A total of 396 patients were included in this study, of whom 227 (57·3 per cent) did not and 169 (42·7 per cent) did receive preoperative oral antibiotics (*Fig*. 1). Some 223 patients (56·3 per cent) were men, and 131 (33·1 per cent) were aged over 65 years. Most had rectal (266 patients, 67·2 per cent) and malignant (90·6 per cent) disease. Laparoscopic resections were carried out in 192 patients (48·5 per cent), with the remainder having open surgery.

Patient characteristics for the entire cohort are shown in *Table* 1. There were significant differences between the group of patients who received preoperative oral antibiotics and MBP and those who did not for age (φ_c_ = 0·124, *P* = 0·048), site of surgery (φ_c_ = 0·199, *P* < 0·001), proportion of malignant disease (φ = −0·372, *P* < 0·001), ASA grade (φ_c_ = 0·217, *P* < 0·001), smoking history (φ_c_ = 0·141, *P* = 0·020), preoperative mGPS (φ_c_ = 0·131, *P* = 0·043), need for intraoperative blood transfusion (φ = −0·135, *P* = 0·008), proportion of patients with a stoma (φ = −0·132, *P* = 0·009) and the use of regional anaesthesia (φ_c_ = 0·391, *P* < 0·001) or dexamethasone (φ = 0·109, *P* = 0·033).

**Table 1:** Association between clinicopathological characteristics, perioperative factors and oral antibiotic administration in patients undergoing left‐sided colonic or rectal surgery, before propensity score matching

	Total (*n* = 396)	No oral antibiotics (*n* = 227)	Oral antibiotics (*n* = 169)	φ/Cramer's V	*P* *
**Age (years)**				0·124	0·048
< 65	183 (46·2)	93 (41·0)	90 (53·3)		
65–74	131 (33·1)	84 (37·0)	47 (27·8)		
≥ 75	82 (20·7)	50 (22·0)	32 (18·9)		
**Sex**				−0·012	0·811
M	223 (56·3)	129 (56·8)	94 (55·6)		
F	173 (43·7)	98 (43·2)	75 (44·4)		
**Site of surgery**				0·199	< 0·001
Left colon	130 (32·8)	65 (28·6)	65 (38·5)		
Rectal (without perineal incision)	219 (55·3)	123 (54·2)	96 (56·8)		
Rectal (with perineal incision)	47 (11·9)	39 (17·2)	8 (4·7)		
**Benign or malignant**	*n* = 395	*n* = 226		−0·372	< 0·001
Benign	37 (9·4)	0 (0)	37 (21·9)		
Malignant	358 (90·6)	226 (100)	132 (78·1)		
**Laparoscopic or open**				0·082	0·101
Laparoscopic	192 (48·5)	102 (44·9)	90 (53·3)		
Open	204 (51·5)	125 (55·1)	79 (46·7)		
**BMI (kg/m** ^**2**^ **)**	*n* = 395	*n* = 226		0·118	0·137
< 20	13 (3·3)	8 (3·5)	5 (3·0)		
20–25	137 (34·7)	86 (38·1)	51 (30·2)		
26–30	137 (34·7)	80 (35·4)	57 (33·7)		
> 30	108 (27·3)	52 (23·0)	56 (33·1)		
**ASA grade**	*n* = 384	*n* = 224	*n* = 160	0·217	< 0·001
I	99 (25·8)	73 (32·6)	26 (16·3)		
II	184 (47·9)	104 (46·4)	80 (50·0)		
III	96 (25·0)	43 (19·2)	53 (33·1)		
IV	5 (1·3)	4 (1·8)	1 (0·6)		
**Smoking status**	*n* = 395	*n* = 226		0·141	0·020
Never	187 (47·3)	109 (48·2)	78 (46·2)		
Ex‐smoker	140 (35·4)	88 (38·9)	52 (30·8)		
Smoker	68 (17·2)	29 (12·8)	39 (23·1)		
**Diabetes**	*n* = 395			0·023	0·649
No	347 (87·8)	200 (88·5)	147 (87·0)		
Yes	48 (12·2)	26 (11·5)	22 (13·0)		
**Preoperative mGPS**	*n* = 367	*n* = 216	*n* = 151	0·131	0·043
0	302 (82·3)	176 (81·5)	126 (83·4)		
1	29 (7·9)	13 (6·0)	16 (10·6)		
2	36 (9·8)	27 (12·5)	9 (6·0)		
**Intraoperative transfusion**	*n* = 385	*n* = 217	*n* = 168	−0·135	0·008
No	372 (96·6)	205 (94·5)	167 (99·4)		
Yes	13 (3·4)	12 (5·5)	1 (0·6)		
**Stoma**				−0·132	0·009
No	204 (51·5)	104 (45·8)	100 (59·2)		
Yes	192 (48·5)	123 (54·2)	69 (40·8)		
**Duration of surgery > 4 h**	*n* = 391	*n* = 225	*n* = 166	0·077	0·128
No	132 (33·8)	83 (36·9)	49 (29·5)		
Yes	259 (66·2)	142 (63·1)	117 (70·5)		
**Other anaesthesia**	*n* = 377	*n* = 211	*n* = 166	0·391	< 0·001
None	85 (22·5)	47 (22·3)	38 (22·9)		
Spinal	144 (38·2)	49 (23·2)	95 (57·2)		
Epidural	98 (26·0)	79 (37·4)	19 (11·4)		
Other	50 (13·3)	36 (17·1)	14 (8·4)		
**Dexamethasone**	*n* = 382	*n* = 215	*n* = 167	0·109	0·033
No	96 (25·1)	63 (29·3)	33 (19·8)		
Yes	286 (74·9)	152 (70·7)	134 (80·2)		

Values in parentheses are percentages. mGPS, modified Glasgow Prognostic Score.

* χ^2^ test.

### Propensity score‐matched patients

**Figure 2:**
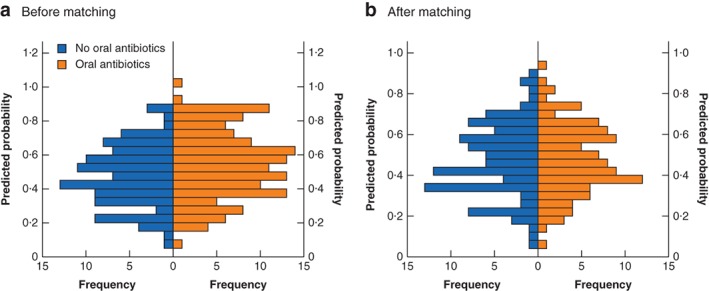
Distribution of propensity scores before and after matching **a** Before and **b** after propensity score matching.

It was not possible to assign propensity scores to 153 patients because of missing co‐variable data; thus, propensity scores were assigned to 243 patients, 102 of whom did not and 141 of whom did receive preoperative oral antibiotics. Overall, 204 patients (102 from each group) were matched based on their propensity score (*Fig*. 1). There was subsequent improvement in the balance of the distribution of propensity scores in each group on visual representation (*Fig*. 2), and improvement in the φ coefficient/Cramer's φ (φ_c_), as shown in *Table* 2.

**Table 2:** Association between clinicopathological characteristics, perioperative factors and oral antibiotic administration in patients undergoing left‐sided colonic or rectal surgery, after propensity score matching

	Variable matched for	Total (*n* = 204)	No oral antibiotics (*n* = 102)	Oral antibiotics (*n* = 102)	φ/Cramer's V
**Age (years)**	Yes				0·114
< 65		91 (44·6)	44 (43·1)	47 (46·1)	
65–74		70 (34·3)	40 (39·2)	30 (29·4)	
≥ 75		43 (21·1)	18 (17·6)	25 (24·5)	
**Sex**	Yes				−0·010
M		115 (56·4)	58 (56·9)	57 (55·9)	
F		89 (43·6)	44 (43·1)	45 (44·1)	
**Site of surgery**	Yes				0·119
Left colon		64 (31·4)	34 (33·3)	30 (29·4)	
Rectal (without perineal incision)		120 (58·8)	55 (53·9)	65 (63·7)	
Rectal (with perineal incision)		20 (9·8)	13 (12·7)	7 (6·9)	
**Benign or malignant**	No				0·271
Benign		14 (6·9)	0 (0)	14 (13·7)	
Malignant		190 (93·1)	102 (100)	88 (86·3)	
**Laparoscopic or open**	Yes				0·010
Laparoscopic		111 (54·4)	55 (53·9)	56 (54·9)	
Open		93 (45·6)	47 (46·1)	46 (45·1)	
**BMI (kg/m** ^**2**^ **)**	Yes				0·038
< 20		4 (2·0)	2 (2·0)	2 (2·0)	
20–25		73 (35·8)	38 (37·3)	35 (34·3)	
26–30		72 (35·3)	36 (35·3)	36 (35·3)	
> 30		55 (27·0)	26 (25·5)	29 (28·4)	
**ASA grade**	Yes				0·077
I		47 (23·0)	25 (24·5)	22 (21·6)	
II		98 (48·0)	50 (49·0)	48 (47·1)	
III		56 (27·5)	25 (24·5)	31 (30·4)	
IV		3 (1·5)	2 (2·0)	1 (1·0)	
**Smoking status**	Yes				0·144
Never		91 (44·6)	43 (42·2)	48 (47·1)	
Ex‐smoker		75 (36·8)	44 (43·1)	31 (30·4)	
Smoker		38 (18·6)	15 (14·7)	23 (22·5)	
**Diabetes**	Yes				0·032
No		182 (89·2)	92 (90·2)	90 (88·2)	
Yes		22 (10·8)	10 (9·8)	12 (11·8)	
**Preoperative mGPS**	Yes				0·018
0		175 (85·8)	88 (86·3)	87 (85·3)	
1		17 (8·3)	8 (7·8)	9 (8·8)	
2		12 (5·9)	6 (5·9)	6 (5·9)	
**Intraoperative transfusion**	Yes				0·000
No		202 (99·0)	101 (99·0)	101 (99·0)	
Yes		2 (1·0)	1 (1·0)	1 (1·0)	
**Duration of surgery > 4 h**	Yes				0·021
No		62 (30·4)	30 (29·4)	32 (31·4)	
Yes		142 (69·6)	72 (70·6)	70 (68·6)	
**Stoma**	Yes				0·020
No		112 (54·9)	57 (55·9)	55 (53·9)	
Yes		92 (45·1)	45 (44·1)	47 (46·1)	
**Other anaesthesia**	Yes				0·300
None		60 (29·4)	39 (38·2)	21 (20·6)	
Spinal		86 (42·2)	28 (27·5)	58 (56·9)	
Epidural		35 (17·2)	21 (20·6)	14 (13·7)	
Other		23 (11·3)	14 (13·7)	9 (8·8)	
**Dexamethasone**	Yes				−0·038
No		37 (18·1)	17 (16·7)	20 (19·6)	
Yes		167 (81·9)	85 (83·3)	82 (80·4)	

Values in parentheses are percentages. mGPS, modified Glasgow Prognostic Score.

In the matched cohort, a significantly smaller proportion of patients who received oral antibiotics had a CRP level of 150 mg/l or above on postoperative day (POD) 4 (17 per cent *versus* 40 per cent in those who did not; *P* < 0·001), but not on POD 3 (35 *versus* 42 per cent respectively; *P* = 0·154) (*Table* 3). Significantly more patients receiving oral antibiotics had a postoperative albumin concentration of 25 mg/l or more on POD 3 (84 *versus* 67 per cent respectively; *P* = 0·003) and POD 4 (78 *versus* 56 per cent; *P* = 0·011) compared with those who did not. Correspondingly, significantly fewer patients receiving oral antibiotics had a poGPS greater than 0 on POD 3 (34 *versus* 43 per cent; *P* = 0·013) and POD 4 (17 *versus* 40 per cent; *P* = 0·001). Corresponding ORs are shown in *Table* 3.

**Table 3:** Patient outcomes within the propensity score‐matched cohort

	Total (*n* = 204)	No oral antibiotics + MBP (*n* = 102)	Oral antibiotics + MBP (*n* = 102)	Odds ratio†	*P*
**CRP ≥ 150 (mg/l) (POD 3)**	*n* = 193	*n* = 95	*n* = 98	0·73 (0·41, 1·31)	0·154
No	119 (61·7)	55 (58)	64 (65)		
Yes	74 (38·3)	40 (42)	34 (35)		
**CRP ≥ 150 (mg/l) (POD 4)**	*n* = 166	*n* = 78	*n* = 88	0·31 (0·15, 0·64)	< 0·001
No	120 (72·3)	47 (60)	73 (83)		
Yes	46 (27·7)	31 (40)	15 (17)		
**Albumin ≥ 25 (mg/l) (POD 3)**	*n* = 190	*n* = 94	*n* = 96	2·66 (1·32, 5·35)	0·003
No	46 (24·2)	31 (33)	15 (16)		
Yes	144 (75·8)	63 (67)	81 (84)		
**Albumin ≥ 25 (mg/l) (POD 4)**	*n* = 167	*n* = 78	*n* = 89	2·67 (1·37, 5·21)	0·011
No	54 (32·3)	34 (44)	20 (22)		
Yes	113 (67·7)	44 (56)	69 (78)		
**poGPS (POD 3)**	*n* = 190	*n* = 94	*n* = 96	0·66 (0·44, 0·99)	0·013
0	117 (61·6)	54 (57)	63 (66)		
1	47 (24·7)	21 (22)	26 (27)		
2	26 (13·7)	19 (20)	7 (7)		
**poGPS (POD 4)**	*n* = 166	*n* = 78	*n* = 88	0·46 (0·30, 0·71)	0·001
0	120 (72·3)	47 (60)	73 (83)		
1	16 (9·6)	8 (10)	8 (9)		
2	30 (18·1)	23 (29)	7 (8)		
**Any complication**				0·31 (0·17, 0·56)	< 0·001
No	120 (58·8)	46 (45·1)	74 (72·5)		
Yes	84 (41·2)	56 (54·9)	28 (27·5)		
**Infective complication**				0·41 (0·22, 0·77)	0·011
No	146 (71·6)	64 (62·7)	82 (80·4)		
Yes	58 (28·4)	38 (37·3)	20 (19·6)		
**SSI**				0·37 (0·17, 0·83)	0·024
No	171 (83,8)	79 (77·5)	92 (90·2)		
Yes	33 (16·2)	23 (22·5)	10 (9·8)		
**Deep SSI**				0·41 (0·10, 1·64)	0·344
No	194 (95·1)	95 (93·1)	99 (97·1)		
Yes	10 (4·9)	7 (6·9)	3 (2·9)		
**Superficial SSI**				0·25 (0·07, 0·93)	0·057
No	190 (93·1)	91 (89·2)	99 (97·1)		
Yes	14 (6·9)	11 (10·8)	3 (2·9)		
**Anastomotic leak**				0·48 (0·14, 1·65)	0·388
No	192 (94·1)	94 (92·2)	98 (96·1)		
Yes	12 (5·9)	8 (7·8)	4 (3·9)		
**Non‐SSI infection**				0·63 (0·27, 1·48)	0·405
No	179 (87·7)	87 (85·3)	92 (90·2)		
Yes	25 (12·3)	15 (14·7)	10 (9·8)		
**Clavien–Dindo complication grade**				0·51 (0·33, 0·80)	0·007
0	123 (60·3)	49 (48·0)	74 (72·5)		
I–II	61 (29·9)	41 (40·2)	20 (19·6)		
III–V	20 (9·8)	12 (11·8)	8 (7·8)		
**Postoperative death**				–	
No	201 (98·5)	99 (97·1)	102 (100)		
Yes	3 (1·5)	3 (2·9)	0 (0)		
**Postoperative length of stay (days)** *		8 (2–122)	7 (2–69)	–	0·050

Values in parentheses are percentages unless indicated otherwise;

* values are median (range);

† values in parentheses are 95 per cent confidence intervals. MBP, mechanical bowel preparation; POD, postoperative day; poGPS, postoperative Glasgow Prognostic Score; SSI, surgical‐site infection.

A significant reduction in overall complications was observed in the group receiving preoperative oral antibiotics (27·5 per cent *versus* 55·9 per cent in patients not receiving oral antibiotics; *P* < 0·001). Preoperative oral antibiotics were associated with a significant reduction in the rate of infective complications (19·6 *versus* 37·3 per cent respectively; *P* = 0·011) and overall SSI (9·8 *versus* 22·5 per cent; *P* = 0·024). A significant reduction was seen in overall Clavien–Dindo complication grade (*P* = 0·007) and postoperative length of stay (7 *versus* 8 days; *P* = 0·050) in those receiving preoperative oral antibiotics. No significant difference was seen with the use of preoperative oral antibiotics and MBP in the rate of deep SSI (2·9 *versus* 6·9 per cent; *P* = 0·344), superficial SSI (2·9 *versus* 10·8 per cent; *P* = 0·057), anastomotic leak (3·9 *versus* 7·8 per cent; *P* = 0·388) or non‐SSI (9·8 *versus* 14·7 per cent; *P* = 0·405). Corresponding ORs are shown in *Table* 3.

Sensitivity analysis of the impact of oral antibiotics and MBP on rates of increased poGPS on POD 3 found a similar statistically significant probability reduction using regression adjustment (OR 0·73, 95 per cent c.i. 0·50 to 1·06) and propensity score matching (OR 0·66, 0·44 to 0·99). Similar results were seen for overall complications using regression adjustment (OR 0·39, 0·23 to 0·66) and propensity score matching (OR 0·31, 0·17 to 0·56), as well as for infective complications: OR 0·48 (0·27 to 0·86) and OR 0·41 (0·22 to 0·77) respectively (*Table* 4).

**Table 4:** Odds ratios for increasing postoperative Glasgow Prognostic Score on day 3, overall and infective complications with respect to use of preoperative oral antibiotics and mechanical bowel preparation across the propensity score methods

		Odds ratio		
	No. of patients	Day 3 poGPS	Overall complications	Infective complications
**Propensity score model**				
Unadjusted	396	0·60 (0·45, 0·80)	0·50 (0·33, 0·75)	0·60 (0·39, 0·94)
PS regression	243	0·73 (0·50, 1·06)	0·39 (0·23, 0·66)	0·48 (0·27, 0·86)
Matched	204	0·66 (0·44, 0·99)	0·31 (0·17, 0·56)	0·41 (0·22, 0·77)

Values in parentheses are 95 per cent confidence intervals. poGPS, postoperative Glasgow Prognostic Score; PS, propensity score.

## Discussion

The addition of preoperative oral antibiotics in combination with MBP to standard ERAS care (including prophylactic intravenous antibiotics) was associated with significantly reduced postoperative systemic inflammatory response, infective complications and length of hospital stay in patients undergoing left‐sided colonic or rectal resection. Within the matched cohort there was a reduced poGPS on POD 3 and 4, and significantly fewer overall complications, overall infective complications, overall SSIs and superficial SSIs. The addition of oral antibiotics improved patient outcomes.

This study complements existing literature supporting the beneficial effect of oral antibiotics in addition to intravenous antibiotics and MBP in reducing postoperative systemic inflammation and complications after left‐sided colonic and rectal resections. This includes a recent international multicentre audit^13^ reporting a reduction in anastomotic leaks with a combination of preoperative oral antibiotics and MBP, and a recent meta‐analysis^15^ suggesting a significant beneficial effect of addition of oral antibiotics to MBP in reducing overall morbidity and mortality, including SSI and anastomotic leak.

The level 1 evidence for preoperative oral antibiotics is limited to a number of relatively small, often historical, trials, as summarized in several recent meta‐analyses^14^, ^15^, ^23^. Many of these trials compared oral antibiotics with no oral antibiotics only in patients receiving MBP. Thus it was not clear whether additional beneficial effects from oral antibiotics would be seen in patients without MBP. The addition of oral antibiotics in patients receiving MBP has, however, now been incorporated into the most recent ERAS® update^5^.

Although not specifically analysed in the present study, the combination of oral antibiotics and MBP was both tolerable and acceptable to patients. Current literature reports minimal adverse events with this intervention. One RCT^24^ reported increased nausea and vomiting in patients receiving three preoperative doses of oral antibiotics compared with that in patients receiving either one dose or no antibiotics (*P* < 0·001), although other trials^25^, ^26^, ^27^, ^28^, ^29^ have reported no negative effects of additional oral antibiotics in terms of enteritis, colitis and/or diarrhoea, including *Clostridium difficile* infection.

A heightened postoperative systemic inflammatory response is associated with both increased postoperative complications and worse long‐term outcomes, including an increased rate of cancer recurrence, poorer survival and decreased quality of life^11^, ^30^, ^31^, ^32^. Reducing both the postoperative systemic inflammatory response and postoperative complications is important in improving both short‐ and long‐term outcomes for the patients, in addition to economic benefits.

The present study has a number of limitations. Propensity score matching to minimize selection bias resulted in the exclusion of a substantial proportion of patients initially included in the study. Although the cohort that did not receive oral antibiotics included only patients undergoing resection for malignant pathology, the group that did receive oral antibiotics and MBP additionally included a small proportion of patients who had a resection for benign pathology. The present analysis did not propensity score match for this variation. It seems unlikely that this discrepancy would have had a significant effect on short‐term outcomes. There was no statistically significant reduction in deep SSI or anastomotic leak rates, as reported in some larger studies^29^. It seems likely that this reflected relatively low rates of these complications that this study was not powered to detect. The longitudinal nature of the study may have introduced bias between groups owing to changes in anaesthetic/surgical technique over the time period included, but no major alterations to surgical or anaesthetic technique, other than the introduction of oral antibiotics and MBP, were apparent. Dexamethasone and regional anaesthesia use were included within propensity score matching to minimize the potential for bias due to these interventions.

The addition of preoperative antibiotics and MBP to standard perioperative care resulted in significant reduction in the postoperative systemic inflammatory response, postoperative morbidity and postoperative length of stay in patients undergoing elective left‐sided colonic or rectal resection. This strategy is worthy of further investigation and potentially wider adoption.

## References

[1] Bray F , Ferlay J , Soerjomataram I , Siegel RL , Torre LA , Jemal A . Global cancer statistics 2018: GLOBOCAN estimates of incidence and mortality worldwide for 36 cancers in 185 countries. CA Cancer J Clin 2018; 68: 394–424. 30207593 10.3322/caac.21492

[2] Cancer Research UK . *Bowel Cancer Incidence Statistics* https://www.cancerresearchuk.org/health-professional/cancer-statistics/statistics-by-cancer-type/bowel-cancer/incidence#heading-Zero [accessed 1 March 2019].

[3] Nelson RL , Gladman E , Barbateskovic M . Antimicrobial prophylaxis for colorectal surgery. Cochrane Database Syst Rev 2014; (5)CD001181. 10.1002/14651858.CD001181.pub4 PMC8406790 24817514

[4] Allegranzi B , Bischoff P , de Jonge S , Kubilay NZ , Zayed B , Gomes SM *et al*; WHO Guidelines Development Group . New WHO recommendations on preoperative measures for surgical site infection prevention: an evidence‐based global perspective. Lancet Infect Dis 2016; 16: e276–e287. 27816413 10.1016/S1473-3099(16)30398-X

[5] Gustafsson UO , Scott MJ , Hubner M , Nygren J , Demartines N , Francis N *et al* Guidelines for perioperative care in elective colorectal surgery: Enhanced Recovery After Surgery (ERAS®) Society Recommendations: 2018. World J Surg 2019; 43: 659–695. 30426190 10.1007/s00268-018-4844-y

[6] McSorley ST , Ramanathan ML , Horgan PG , McMillan DC . Postoperative C‐reactive protein measurement predicts the severity of complications following surgery for colorectal cancer. Int J Colorectal Dis 2015; 30: 913–917. 25922147 10.1007/s00384-015-2229-3

[7] Singh PP , Zeng IS , Srinivasa S , Lemanu DP , Connolly AB , Hill AG . Systematic review and meta‐analysis of use of serum C‐reactive protein levels to predict anastomotic leak after colorectal surgery. Br J Surg 2014; 101: 339–346. 24311257 10.1002/bjs.9354

[8] Watt DG , McSorley ST , Park JH , Horgan PG , McMillan DC . A postoperative systemic inflammation score predicts short‐ and long‐term outcomes in patients undergoing surgery for colorectal cancer. Ann Surg Oncol 2017; 24: 1100–1109. 27822634 10.1245/s10434-016-5659-4

[9] Watt DG , McSorley ST , Horgan PG , McMillan DC . Enhanced recovery after surgery: which components, if any, impact on the systemic inflammatory response following colorectal surgery?: a systematic review. Medicine (Baltimore) 2015; 94: e1286. 26356689 10.1097/MD.0000000000001286 PMC4616657

[10] Dolan RD , Lim J , McSorley ST , Horgan PG , McMillan DC . The role of the systemic inflammatory response in predicting outcomes in patients with operable cancer: systematic review and meta‐analysis. Sci Rep 2017; 7: 16717. 29196718 10.1038/s41598-017-16955-5 PMC5711862

[11] Artinyan A , Orcutt ST , Anaya DA , Richardson P , Chen GJ , Berger DH . Infectious postoperative complications decrease long‐term survival in patients undergoing curative surgery for colorectal cancer: a study of 12 075 patients. Ann Surg 2015; 261: 497–505. 25185465 10.1097/SLA.0000000000000854

[12] Devane LA , Proud D , O'Connell PR , Panis YA . European survey of bowel preparation in colorectal surgery. Colorectal Dis 2017; 19: O402–O406. 28975694 10.1111/codi.13905

[13] 2017 European Society of Coloproctology (ESCP) Collaborating Group . Association of mechanical bowel preparation with oral antibiotics and anastomotic leak following left sided colorectal resection: an international, multi‐centre, prospective audit. Colorectal Dis 2018; 20(Suppl 6): 15–32. 30255646 10.1111/codi.14362

[14] McSorley ST , Steele CW , McMahon AJ . Meta‐analysis of oral antibiotics, in combination with preoperative intravenous antibiotics and mechanical bowel preparation the day before surgery, compared with intravenous antibiotics and mechanical bowel preparation alone to reduce surgical‐site infections in elective colorectal surgery. BJS Open 2018; 2: 185–194. 30079387 10.1002/bjs5.68 PMC6069350

[15] Rollins KE , Javanmard‐Emamghissi H , Acheson AG , Lobo DN . The role of oral antibiotic preparation in elective colorectal surgery: a meta‐analysis. Ann Surg 2019; 270: 43–58. 30570543 10.1097/SLA.0000000000003145 PMC6570620

[16] Toh JWT , Phan K , Hitos K , Pathma‐Nathan N , El‐Khoury T , Richardson AJ *et al* Association of mechanical bowel preparation and oral antibiotics before elective colorectal surgery with surgical site infection: a network meta‐analysis. JAMA Netw Open 2018; 1: e183226. 30646234 10.1001/jamanetworkopen.2018.3226 PMC6324461

[17] Chen M , Song X , Chen LZ , Lin ZD , Zhang XL . Comparing mechanical bowel preparation with both oral and systemic antibiotics *versus* mechanical bowel preparation and systemic antibiotics alone for the prevention of surgical site infection after elective colorectal surgery: a meta‐analysis of randomized controlled clinical trials. Dis Colon Rectum 2016; 59: 70–78. 26651115 10.1097/DCR.0000000000000524

[18] Eslami M , Yousefi B , Kokhaei P , Hemati M , Nejad ZR , Arabkari V *et al* Importance of probiotics in the prevention and treatment of colorectal cancer. J Cell Physiol 2019; 234: 17 127–17 143. 10.1002/jcp.28473 30912128

[19] Schirmer M , Smeekens SP , Vlamakis H , Jaeger M , Oosting M , Franzosa EA *et al* Linking the human gut microbiome to inflammatory cytokine production capacity. Cell 2016; 167: 1897. 10.1016/j.cell.2016.11.046 27984736

[20] McMillan DC . The systemic inflammation‐based Glasgow Prognostic Score: a decade of experience in patients with cancer. Cancer Treat Rev 2013; 39: 534–540. 22995477 10.1016/j.ctrv.2012.08.003

[21] Dindo D , Demartines N , Clavien PA . Classification of surgical complications: a new proposal with evaluation in a cohort of 6336 patients and results of a survey. Ann Surg 2004; 240: 205–213. 15273542 10.1097/01.sla.0000133083.54934.ae PMC1360123

[22] NCSS Statistical Software . *PASS Sample Size Software: Tests for Multiple Correlated Proportions (McNemar–Bowker Test of Symmetry)* https://ncss-wpengine.netdna-ssl.com/wp-content/themes/ncss/pdf/Procedures/PASS/Tests_for_Multiple_Correlated_Proportions-McNemar-Bowker_Test_of_Symmetry.pdf [accessed 1 August 2019].]

[23] Koullouros M , Khan N , Aly EH . The role of oral antibiotics prophylaxis in prevention of surgical site infection in colorectal surgery. Int J Colorectal Dis 2017; 32: 1–18. 27778060 10.1007/s00384-016-2662-y

[24] Espin‐Basany E , Sanchez‐Garcia JL , Lopez‐Cano M , Lozoya‐Trujillo R , Medarde‐Ferrer M , Armadans‐Gil L *et al* Prospective, randomised study on antibiotic prophylaxis in colorectal surgery. Is it really necessary to use oral antibiotics? Int J Colorectal Dis 2005; 20: 542–546. 15843938 10.1007/s00384-004-0736-8

[25] Hata H , Yamaguchi T , Hasegawa S , Nomura A , Hida K , Nishitai R *et al* Oral and parenteral *versus* parenteral antibiotic prophylaxis in elective laparoscopic colorectal surgery (JMTO PREV 07‐01): a phase 3, multicenter, open‐label, randomized trial. Ann Surg 2016; 263: 1085–1091. 26756752 10.1097/SLA.0000000000001581

[26] Ikeda A , Konishi T , Ueno M , Fukunaga Y , Nagayama S , Fujimoto Y *et al* Randomized clinical trial of oral and intravenous *versus* intravenous antibiotic prophylaxis for laparoscopic colorectal resection. Br J Surg 2016; 103: 1608–1615. 27550722 10.1002/bjs.10281

[27] Uchino M , Ikeuchi H , Bando T , Chohno T , Sasaki H , Horio Y *et al* Efficacy of preoperative oral antibiotic prophylaxis for the prevention of surgical site infections in patients with Crohn disease: a randomized controlled trial. Ann Surg 2019; 269: 420–426. 29064884 10.1097/SLA.0000000000002567

[28] Ozdemir S , Gulpinar K , Ozis SE , Sahli Z , Kesikli SA , Korkmaz A *et al* The effects of preoperative oral antibiotic use on the development of surgical site infection after elective colorectal resections: a retrospective cohort analysis in consecutively operated 90 patients. Int J Surg 2016; 33: 102–108. 27463886 10.1016/j.ijsu.2016.07.060

[29] Klinger AL , Green H , Monlezun DJ , Beck D , Kann B , Vargas HD *et al* The role of bowel preparation in colorectal surgery: results of the 2012–2015 ACS‐NSQIP Data. Ann Surg 2019; 269: 671–677. 29064902 10.1097/SLA.0000000000002568

[30] Oh SY , Kim YB , Suh KW . Prognostic significance of systemic inflammatory response in stage II colorectal cancer. J Surg Res 2017; 208: 158–165. 27993203 10.1016/j.jss.2016.08.100

[31] McSorley ST , Watt DG , Horgan PG , McMillan DC . Postoperative systemic inflammatory response, complication severity, and survival following surgery for colorectal cancer. Ann Surg Oncol 2016; 23: 2832–2840. 27016295 10.1245/s10434-016-5204-5 PMC4972846

[32] Brown SR , Mathew R , Keding A , Marshall HC , Brown JM , Jayne DG . The impact of postoperative complications on long‐term quality of life after curative colorectal cancer surgery. Ann Surg 2014; 259: 916–923. 24374539 10.1097/SLA.0000000000000407

